# The Book World of Medicine and Science

**Published:** 1906-02-17

**Authors:** 


### The Book World of Medicine and Science.

Treatise on Orthopedic Surgery. Bradford and Lovett. Third edition. Baillière, Tindall and Cox, pp. 670. Illustrations 592. Price 21s. net.

An eminently practical book on their own subject comes from the Professor of Orthopædic Surgery in Harvard Medical School and his assistant. The enthusiasm for efficiency of treatment, wherein lies the secret of success in orthopædics, is the centre of the authors' argument, which is enforced by a strong and vigorous style. The chapters on the treatment of tuberculosis of bones and joints are full of practical suggestions, and express the conviction that, while early and thorough treatment are desirable, much can be done, even in advanced cases of deformity, for the alleviation of the patient. The description of lateral curvatures and their treatment is particularly worthy of perusal. In connection with anterior poliomyelitis a much needed word of warning is given against allowing paralysed limbs to lie in bad position at the onset of the disease. "Stretched muscles are notoriously at a disadvantage, so far as recovery goes, in any diseased condition, and muscles at rest are much more favourably situated. So that this very point may determine in a measure the relative amount of recovery in the two groups" (*i.e.*, anterior and posterior muscle groups of the lower limb). "It is highly important to support and restrain the limb in a normal position." While in scope both as to conditions described and their treatment the book fulfils the requirements of a text-book on orthopædics, there are one or two points in treatment to which exception may be taken. We do not agree that "excision of the elbow is indicated earlier in the course of tubercular disease than is the case in any other of the larger joints," nor do we admit that "ankylosis is aimed at as the best possible result" in this joint. The authors advocate treatment of the elbow in the right-angled position, and do not refer to the treatment in the fully flexed position recommended by Jones of Liverpool and others. We have found that treatment in the latter position leads to a recovery with a good range of movement in a large number of cases, even where the disease is advanced. The value of this edition has been much enhanced by the addition of a chapter on practical details of apparatus, in which not merely the necessary measurements are described, but also the materials and the strength desirable in the various parts, and in many instances an estimate of the cost of materials. Following the instructions of this chapter the surgeon may easily have almost any of the apparatus referred to in the text turned out at small cost by any intelligent blacksmith. The illustrations are nearly all from photographs and have been carefully chosen to indicate the features of disease referred to in the text. With so much to praise and so little to call in question, the book is one which can be commended for careful study by the surgeon and almost more so by the practitioner.

Lectures on Clinical Psychiatry. By Dr. Emil Kraepelin, Professor of Psychiatry in the University of Munich. Revised and edited by Thomas Johnstone, M.D.Edin., M.R.C.P.Lond. (London: Baillière, Tindall and Cox. Pp. 352. Price 10s. 6d. net.)

We reviewed the first edition of this book in 1904, and we then expressed a high opinion of the work as an addition to medico-psychological literature, giving also a sample of the admirable manner in which Dr. Johnstone had done his work as translator and editor. The fact that a second edition of a work on such a special subject has already been called for shows that our opinion of the book has been corroborated by the profession at large. This, the second English edition, has been rendered more instructive by the addition of two fresh lectures: one on "Congenital States of Diseases," and the other on "Morbid Criminals and Vagabonds." Both of these lectures possess the same characteristics as the whole of the older work in being true living pictures of disease and of those forms of insanity which so often place their victims in direct antagonism to the law and to society. Perhaps these lectures may help the medical jurist to trace that very elusive line of demarcation between crime and insanity. The author in his preface to this new edition tells us that he has substituted more appropriate examples than some of those used in the first edition, and that regarding the lecture on insanity of degeneration he has added seven new cases. The size of the work has been increased from 308 pages to 352, and, while its usefulness has been added to, its bulk is yet by no means unwieldy.

Departmental Decisions.

Under the title of Departmental Decisions the first instalment of what promises to be a useful publication, and is to be continued quarterly, has just been issued by the management of the "Local Government Journal." It contains, extracted from the journal, the decisions of eight Government departments: the Board of Education, the Local Government Board, the Board of Trade, the Treasury, the Home Office, the War Department, the Admiralty, and the Post Office, concerning questions which have been submitted to them in correspondence by local authorities, or have been answered in Parliament. The first issue covers the last quarter of 1905, and contains 203 decisions; those of the Board of Education and of the Local Government Board contributing 191 of the total, and pointing alike to the difficulties of administering the Education Act and to the number and complexity of the interests which are committed to the last-named department. As the decisions quoted will be precedents, it is possible that an easily accessible record may often be of great convenience to officials; and the little book in one respect recalls the old lady's dscription of Johnson's Dictionary. The narrative is certainly unconnected, even if we admit the language to be deserving of praise.

Local Government Annual, 1906. (London: The "Local Government Journal," Limited, 27a Farringdon Street, E.C. Price 1s. 6d.)

This very useful annual contains official particulars concerning the various Local Government bodies throughout the United Kingdom. The work appears to be very complete, and should prove exceedingly valuable to all requiring such information.

